# Editorial: Web Tools for Modeling and Analysis of Biomolecular Interactions

**DOI:** 10.3389/fmolb.2022.875859

**Published:** 2022-04-11

**Authors:** Jessica Andreani, Masahito Ohue, Brian Jiménez-García

**Affiliations:** ^1^ Université Paris-Saclay, CEA, CNRS, Institute for Integrative Biology of the Cell (I2BC), Gif-sur-Yvette, France; ^2^ Department of Computer Science, School of Computing, Tokyo Institute of Technology, Kanagawa, Japan; ^3^ Zymvol Biomodeling, Barcelona, Spain

**Keywords:** web tools, biomolecular interactions, macromolecular structure, macromolecular evolution, protein docking, protein-ligand complexes, visualization, web server

This Research Topic presents recent (or recently updated) web-based tools and databases in the field of computational modeling and analysis of biomolecular interactions. Over the past few decades, web-based tools and databases have flourished thanks to their wide availability and ease of use. They offer several advantages over stand-alone tools, such as not requiring special skills for installing them, and ubiquity (as only a web browser is needed to access and use them). Importantly, they also represent an honest attempt to democratize science. In addition, when well identified and referenced, these tools and databases have the potential to be interconnected and enriched through their composition as building blocks.

The field of computational modeling and analysis of biomolecular interactions is extremely active. Those interactions are of the utmost importance in living organisms and being able to analyze and predict such interactions is extremely relevant, from basic biological research to possible applications in drug developments or molecular systems engineering. A wealth of experimental data has recently become available thanks to the development of high-throughput techniques, making data integration a crucial challenge to exploit them. Additionally, recent evolutions in machine and deep learning-based methods have opened new perspectives for the development of predictive strategies.

Computational modeling and analysis of biomolecular interactions is thus a relevant and timely topic for biologists. The necessity to provide accessible methods for non-computational users makes web-based tools particularly attractive and interesting to a wide audience in molecular biosciences. It is therefore of the utmost importance, and it was our purpose in this Research Topic, to promote web tools for modeling and analysis of biomolecular interactions that are well documented, supported, available online freely for academics and as easy to use as possible for non-specialists.

In this Research Topic Editorial, we arranged the publications by the type of biomolecular interactions addressed by each tool, as well as the type of data. The majority of papers in this Topic present web tools related to 3D structure of biomolecules and their complexes, although a few of them (summarized at the end of this editorial) address complementary questions such as protein localization, high-throughput omics data analysis and systems biology simulations.

Four papers present tools for the modeling and analysis of protein-protein complexes. Two of them present protein docking servers: DeepComplex and LZerD. DeepComplex (Quadir et al.) is an automated web server for predicting protein complex structures with deep learning techniques. It uses machine learning to predict inter-chain contacts in a homodimer or heterodimer form and the predicted contacts are then used to construct a quaternary structure of the dimer by distance-based modeling, which can be interactively viewed and analyzed. LZerD is a veteran web server for macromolecular complex prediction, which presents in this Research Topic several updates (Christoffer et al.), including a new functionality of *de novo* prediction of subunit structures by the recently published AttentiveDist method, and applying symmetry constraints for homodimer modeling. Another server, QSalignWeb (Dey et al.), is dedicated to the prediction and analysis of quaternary structure states of protein homo-oligomers, allowing users to submit their own structures to the QSalign pipeline, which makes use of evolutionary conservation to predict physiologically relevant protein assemblies. Finally, Barreto et al. presents a database dedicated to the analysis of protein interactions between G-protein coupled receptors (GPCRs) of the opioid family and their partners involves the integration of multiple modeling strategies to uncover key structural determinants involved in GPCR interaction specificity.

Three papers then address the topic of protein-ligand complexes. Fuzzle 2.0 (Ferruz et al.) is a recent update of an evolution-related database to identify and analyze conserved parts of a protein. In the version 2.0 of the Fuzzle database, protein fragments binding to specific ligands can be also identified and analyzed. PreBINDS (Ikeda et al.) is a web tool to build datasets for compound-protein interaction (CPI) prediction with supervised machine learning. Via the web server interface, users can customize the CPI dataset derived from ChEMBL by setting positive and negative thresholds to be adjusted according to the user definitions. SeamDock (Murail et al.) aims at providing a free and accessible protein-ligand (small molecule) docking tool, in particular for teaching purposes. SeamDock’s ease of use combined with a complete 3D visualization in a collaborative mode makes it a perfect tool for non-specialists outside of the molecular modeling community.

Two papers describe web tools for visualization and analysis of 3D structures. 3dRS (Bayarri et al.) is an online tool with the goal of sharing biomolecular structure representations, including molecular dynamics trajectories. 3dRS provides an unique URL to share and discuss structural data in an interactive fashion, including the possibility to use it as a live figure for scientific papers. VTR (Pimentel et al.) is a novel approach with a visual web interface that can be used to analyze, compare, and scrutinize analogous contacts in protein pairs. VTR can be used for understanding differences and similarities between homologous proteins with similar 3D structures but differences in sequences.

Two more papers complement this Research Topic of web tools related to 3D structures of biomolecules. SHAPER (Zhou et al.) is a fast and accurate web server for predicting SHAPE (selective 2′-hydroxyl acylation analyzed by primer extension) probing reaction profile for any given RNA structure, providing information about the local flexibility of RNA. An overview paper presents the WeNMR-EOSC (Honorato et al.) collection of web services for structural biology workflows, including experimental NMR, cryoEM, crosslinking-mass spectrometry data analysis, docking and several more; this computational infrastructure has been successfully running for over 10 years and it is part of the European Open Science Cloud portal, overall reaching a wide community of users.

Last but not least, three non-structural papers complete this Research Topic. The SGnn web server (Iglesias et al.) can be used to predict the recruitment of prion-like domain sequences in proteins to condensates, specifically stress granules, on the basis of biophysical properties used as features in a neural network model. GeneTrail (Gerstner et al.), a suite of web tools, allows the user to analyze and visualize various types of “omics” profiles in an integrative approach, taking data from bulk, time-series, or single-cell experiments. The online GeneTrail analysis aims to identify deregulated biological processes by comparing disease with control data and to propose candidates that might drive this deregulation. Finally, the WebMaBoss (Noël et al.) web interface enables easy manipulation of Boolean models and further simulation of Boolean networks, such as biological networks involved in gene regulation or signaling, followed by online visualization and analysis.

Overall, this Research Topic represents the effort of the computational modeling and analysis of biomolecular interactions field to provide well-designed web-based tools to the community. All the tools presented here are actively developed and well documented, with the intention of enriching the scientific tools landscape, and freely accessible through the World Wide Web. Science is collaborative and web-based tools and databases are the pillars of open science. As the editors of this Research Topic, we would like to thank all researchers contributing to this Research Topic for their engagement towards Open Science best values.

